# Radiologic evaluation of orbital index among Ghanaians using CT scan

**DOI:** 10.1186/s40101-017-0145-7

**Published:** 2017-07-11

**Authors:** Benard Ohene Botwe, Derick Seyram Sule, Abdul Mumin Ismael

**Affiliations:** Department of Radiography, School of Biomedical and Allied health Sciences, University of Ghana, Accra, Ghana

**Keywords:** Orbital index, Ghanaian, Microseme, Forensic, CT

## Abstract

**Background:**

Orbital index (OI) expresses the proportion of the orbital height to the orbital width and varies with race, regions within the same race and periods in evolution. This index is useful in forensic medicine, anthropology and surgery. However, the average OI among Ghanaian adults was unknown.

**Aim:**

The aim of this study was to determine the orbital index of adult Ghanaians and classify them under one of the three predetermined groups.

**Method:**

The study design was a retrospective cross-sectional. A systematic random sampling method was used for selecting 350 adult Ghanaian head computed tomography images available from 1 January to 31 December 2015 at KBTH Hospital. The orbital height and orbital width of each orbit were measured on a 3D CT skull. Data was analysed using Microsoft Excel and Statistical Package for Social Sciences version 20.

**Results:**

The study had more females than men (167, 47.71%, vs 183, 52.29%). The observed orbital index of Ghanaians in the study was 81.22 ± 4.22. The mean orbital index was 80.52 ± 4.66 in males and 82.15 ± 3.83 in females with their difference being statistically significant (*p* value <0.05). This placed both genders in the Microseme category of orbit. There was no significant difference between the orbital index of the two orbital sides (left and right orbits).

**Conclusion:**

The study found Ghanaians in the category of the Microseme and also indicated a strong sexual dimorphism. The outcome of this study may be useful in forensic medicine for skull classification and also for better surgical approach in neurosurgery as well as cosmetic surgery.

## Background

The determination of origin and identity of the skeletal remains collected from a crime scene is an important and difficult task. Different craniofacial measurements and indices can be useful for this purpose. The mode of determining the parameters needed for the estimation of these indices depends on the type of samples used. However, dry bone collection with all the information available about the bones has been indicated to be the best source of sample to work on [1]. However, advanced radiological techniques have been proven to be the better choice in cases where skull collection is a problem [2].

Several craniofacial indices have been used in the determination of population origin and identity. An example is the orbital index (OI) which involves taking measurement between various landmarks on the orbit. The orbital cavities are located on the opposite sides of the midsagittal plane of the skull and fall at a point between the cranium and the facial bones [3]. These cavities are each intended to serve as a socket for the eye balls and also contain associated muscles, vessels, nerves, lacrimal apparatus, facial strata and soft pad [4]. Each bony orbit is composed of seven bones: maxilla, palatine, frontal, zygoma, sphenoid, ethmoid and lacrimal. These bones are arranged to enclose a roughly quadrilateral pyramidal cavity. Among modern human groups, the characteristics of the orbit vary considerably [5].

Among parameters estimated during the craniofacial morphometric examination is the OI, the proportion of the orbit height to its width multiplied by 100%. This is known to be determined by the shape of the face and varies with race, regions within the same race and periods in evolution [6]. The OI has been studied by many authors. Ezeuko and Om’Iniabohs [7] evaluated the OI among the Igbo ethnic group of Nigeria. Igbigbi and Ebite [8] recorded the OI of the Malawian to be part of the Megaseme category. Also, Kaur et al. [3] reported Microseme type of OI in the Bathinda population of India. All these studies indicated some level of racial and ethnic variation in the OI of various population groups.

The knowledge of this index is therefore very applicable in various fields such as in interpretation of fossil records, skull classification in forensic medicine and in exploring the trends in evolutionary and ethnic differences. Furthermore, documented ranges of this index in different nationalistic groups will assist in skull identification [7], in particular, among difference races, where forensic data is not available. In addition, a prior knowledge of the orbital morphometry is very essential for better surgical approach and outcome. Countries like Japan, China, India, Malawi and many others have classified their population under one of the above predetermined categories (i.e. Megaseme, OI = 89 or over; Mesoseme, OI = 89–83; and Microseme, OI = 83 or less), based on their average OI. However, there is no study in literature pertaining to morphometry of orbit in Ghanaian population. Therefore, this study of orbital morphometry in the skulls of Ghanaians has developed a database to determine normal range of orbital values and OI in the Ghanaian population.

## Method

A retrospective quantitative cross-sectional approach was adopted for the study. The study was conducted at the Computed Tomography Unit of the Korle-Bu Teaching Hospital, where computed tomography (CT) data of patients are stored. This hospital is the biggest Hospital in Ghana which receives people (patients) from all parts of country. Systematic random sampling method was used to select 350 normal adult (18 years and above) head CT scans of Ghanaians, who had reported to the CT Unit for head CT scan from 1 January to 31 December 2015. Prior the sampling, CT scan images of subjects with craniofacial abnormality and prior craniofacial surgery were excluded. In addition, CT images from foreign nationals were not included in the study.

**Fig. 1:**
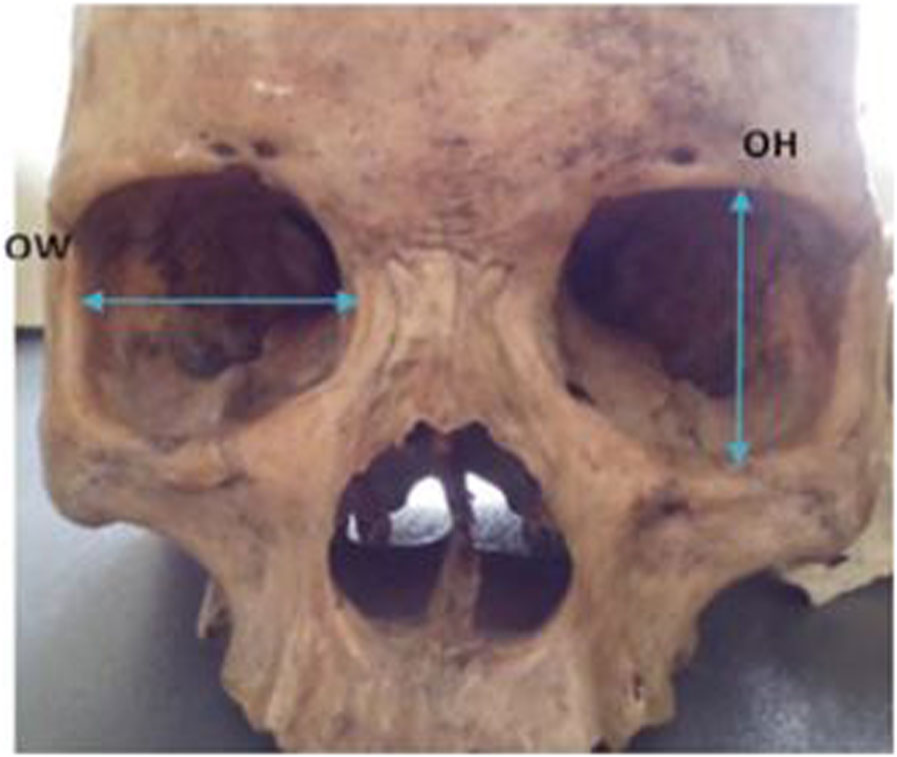
Showing orbital dimensions (OH and OW)

In the study, all volumetric head CT scan images which were obtained within the specified period of the study and sampled from the backup system of the CT unit were converted into 3D version. Measurements were done using the computer software of the Toshiba Aquillion ONE V4.82 ER001 640 slices CT equipment and a simple and brief data collection spread sheet was designed to record the data. The orbital width (OW) (the distance between the dacryon to orbital tubercle) and orbital height (OH) (the distance between superior and inferior margin at the midpoint and perpendicular to the OW), as depicted in Fig. 1, were recorded for each subject. In all, the data collected included age, gender, OH and OW.

The formula $\mathrm{O}\mathrm{I} = \frac{\mathrm{OH}}{\mathrm{OW}}\times 100\%$ was used to determine the OI.

Microsoft excel and Statistical Package for Social Sciences (SPSS) version 20 was used to analyse the data. Microsoft excel was used to generate tables and graphs, respectively. Independent sample *t* test and paired *t* test were used to determine the statistical significance of all parameters of orbits, with respect to gender and side (right and left side) respectively. ANOVA was also used to determine the significance of the variation between the orbital indices of various age groups. The results were considered significant when *p* value <0.05 and were considered highly significant when *p* value <0.001. The results are reported as mean, standard deviation, mean difference and *p* value in the form of tables and graphs.

In accordance with the established protocols on research, ethical approval was sought from the Ethical and Protocol Review Committee of the College of Health Sciences of University of Ghana for the commencement of this study. Permission was also sought from the Head of the Radiology Department of the Korle-Bu Teaching Hospital for access to the stored data. Patients’ anonymity and confidentiality were in respect of ethics ensured appropriately.

In accordance with the established protocols on research of the University of Ghana, ethical approval was sought from the Ethical and Protocol Review Committee of the College of Health Sciences of the University of Ghana for the commencement of this study. Permission was also sought from the Head of the Radiology Department of the Korle-Bu Teaching Hospital for access to the stored data. In addition, codes were assigned to the CT images and were also stored using a hard drive on the researcher’s laptop locked with a password which is known by only the researcher. These helped in ensuring patient’s anonymity and confidentiality.

## Results

**Fig. 2:**
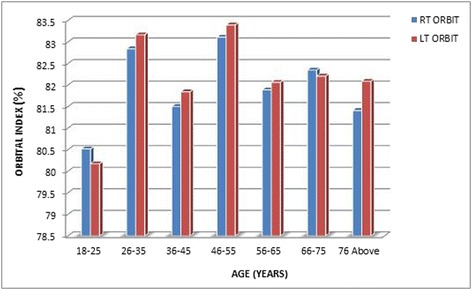
OI of the various female age groups with respect to the orbital sides

**Fig. 3:**
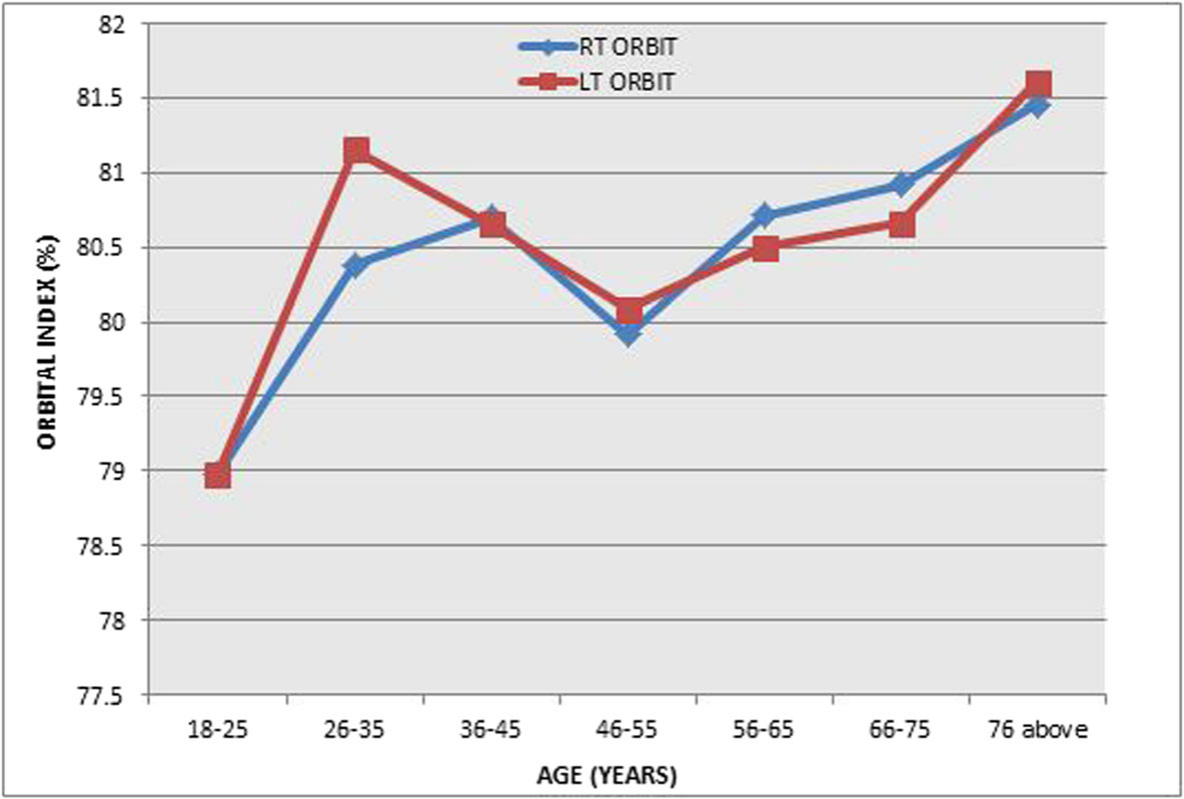
OI of the various male age groups with respect to the orbital sides

Three hundred and fifty (350) head CT scan images were used for the study which consisted of 167 female images (47.71%) and 183 male images (52.29%). The age range was 18–93 years. The range of observed orbital height was 30.8–41.2 mm which varies 30.8–41.2 mm in females and 31.3–40.3 mm in males. Also, the range of observed orbital width was 40.0–48.2 mm in females and 40.0–48.7 mm in males. The general range of OW was also observed to be 40.0–48.7 mm. By using OH and width, the OI was calculated. The range of observed OI was 71.86–93.7 in females and 70.02–93.99 in males. Thus, the general observed range of OI was 70.02–93.99 with a mean OI being 81.22 ± 4.22 for the study population. The comparisons between male and female indices, left and right orbital dimensions and the left and right orbital indices of the two gender groups are presented in Tables 1, 2 and 3, respectively. In addition, the comparisons between OI of age groups with respect to the orbital sides of female, and OI of male age groupings with respect to the orbital sides, are presented in Tables 4 and 5. Figures 2 and 3 also present the comparison results of the orbital index of the various age groups of the female and male with respect to the orbital sides. Findings of this study in terms of orbital index of the orbital sides, and that of OI among the genders, were compared to other studies, and the findings are shown in Table 6 and 7, respectively.

**Table 1:** Comparison between male and female orbital indices

Parameters	Gender	Mean	SD	Mean difference	*p* value
Height (mm)	Female	35.14	1.69	0.13	0.35
	Male	35.01	1.92		
Width (mm)	Female	42.81	1.58	−0.72	0.00
	Male	43.53	1.78		
OI (%)	Female	82.15	3.83	1.63	0.00
	Male	80.52	4.66

**Table 2:** Comparison between left and right orbital dimensions

Parameter	Side	Mean	S D	Mean difference	*p* value
Height (mm)	Left	35.08	1.83	0.01	0.71
	Right	35.07	1.80		
Width (mm)	Left	43.15	1.74	−0.06	0.018
	Right	43.21	1.71		
OI (%)	Left	81.37	4.48	0.15	0.221
	Right	81.22	4.24

**Table 3:** Comparison between the left and right orbital indices of the two gender groups

Side	Gender	Mean (%)	S D	Mean difference	*p* value
RTOI	Female	82.06	3.86	1.60	0.00
	Male	80.45	4.43		
LTOI	Female	82.24	3.80	1.66	0.00
	Male	80.58	4.90

**Table 4:** Comparison between OI of age groups with respect to the orbital sides of female

Side	Age (years)	Frequency	Mean (%)	SD	Minimum (%)	Maximum (%)	*p* value
Right	18–25	17	80.5202	2.55947	75.64	85.27	0.342
	26–35	30	82.8447	2.77967	77.98	88.65	
	36–45	27	81.5057	4.11318	71.86	90.82	
	46–55	22	83.1148	5.48581	72.99	93.70	
	56–65	26	81.8915	3.32048	76.75	88.57	
	66–75	29	82.3514	4.05725	76.39	89.88	
	76 Above	16	81.4123	3.98478	76.70	89.25	
	Total	167	82.0559	3.86280	71.86	93.70	
Left	18–25	17	80.1755	2.73002	74.94	85.65	0.159
	26–35	30	83.1695	3.15333	78.03	89.05	
	36–45	27	81.8468	4.03823	74.66	91.02	
	46–55	22	83.3973	4.99640	75.00	93.20	
	56–65	26	82.0666	3.36512	76.52	88.00	
	66–75	29	82.2109	3.78118	75.45	89.44	
	76 above	16	82.0903	3.84414	77.53	89.93	
	Total	167	82.2393	3.79768	74.66	93.20

**Table 5:** Comparison between OI of male age groupings with respect to the orbital sides

Side	Age (years)	Frequency	Mean (%)	SD	Minimum (%)	Maximum (%)	*p* value
Right	18–25	19	78.9770	4.60167	71.12	88.66	0.679
	26–35	37	80.3821	4.90736	70.44	93.72	
	36–45	25	80.6998	4.44213	73.23	88.97	
	46–55	27	79.9174	3.55092	71.86	87.62	
	56–65	38	80.7150	4.41730	72.05	91.08	
	66–75	15	80.9165	6.12256	72.16	93.99	
	76 above	22	81.4567	3.00702	75.12	87.81	
	Total	183	80.4532	4.42584	70.44	93.99	
Left	18–25	19	78.9782	4.33440	70.02	88.69	0.709
	26–35	37	81.1583	7.18298	70.59	112.94	
	36–45	25	80.6563	4.22157	74.38	89.50	
	46–55	27	80.0834	3.98571	71.96	88.83	
	56–65	38	80.4962	4.26004	72.05	90.67	
	66–75	15	80.6611	5.50559	71.24	89.05	
	76 above	22	81.6081	2.68264	76.12	84.80	
	Total	183	80.5806	4.90282	70.02	112.94

**Table 6:** Comparison of orbital index of the orbital sides to other studies

Author	Right	Left	*p* value
Mekala et al. [10]	85.22 ± 7.21	84. 2± 7.24	0.716
Ezeuko and Om’Iniabohs [7]	72.20 ± 13.5	72.00 ± 13.1	0.88
Gopalakrishna and Kashinatha [12]	80.69 ± 2.19	81.16 ± 2.02	0.011
Present study	81.22 ± 4.24	81.37 ± 4.48	0.22

**Table 7:** Comparison of OI in present study with other studies among the genders

Author	Male	Female	P-value	Category
Ezeuko and Om’Iniabohs [7]	73.6 ± 13.3	69.9 ± 13.5	0.014	Microseme
Ebeye and Otikpo [11]	78.15 ± 0.82	78.57 ± 0.6	<0.05	Microseme
Mekala et al. [10]	84.62 ± 8.21	85.46 ± 5.93	0.14	Mesoseme
Present study	80.52 ± 4.66	82.15 ± 3.83	0.00	Microseme

## Discussion

The orbit is a craniofacial structure located on the anterior surface of the skull. Its morphology varies considerably between race and ethnic groups belonging to different geographical regions of the world. This study was aimed at determining the orbital dimensions and OI of Ghanaians who reported to the radiology unit of KBTH. The knowledge of these orbital dimensions and index is applicable in skull classification in forensic medicine and also very essential for better surgical approach and outcome. As such, various orbital measurement were taken on a 3D bone reconstructed images which, according to Cavalcanti et al. [9], is highly accurate and deemed a better choice when skeletal remains are not available for direct measurement.

The mean OI of the female and male were found to be 82.15 ± 3.83 and 80.52 ± 4.66, respectively. These values placed both genders in the Microseme category of orbit. This confirms a previous study which demonstrated that the black races have Microseme OI [10].

Comparison between the mean OI of the two genders also showed a significantly higher OI in females than in the males. This is in agreement with Ezeuko and Om’Iniabohs [7] and Ebeye and Otikpo [11] who documented similar finding between the two genders. However, unlike Ebeye and Otikpo [11], Ezeuko and Om’Iniabohs [7] found the male OI to be significantly higher in males than that of the females. With regards to the difference between the OI of the genders, the result of this current study was in contrast with the findings of Mekala et al. [10].

Moreover, comparison between the dimensions of left and right orbits shows that the difference between the mean OH and OW of both orbital sides was statistically not significant, indicating that the orbit with respect to sides have similar dimensions.

When the mean OI of both sides (right and left) were compared, the difference was also statistically not significant. This is in line with the findings of Mekala et al. [11] and Ezeuko and Om’Iniabohs [7]. However, the findings of this study contradict that of Gopalakrishna and Kashinatha [12].

Notwithstanding, a statistically significant value was observed when both right and left OI were compared with respect to the gender groups, to indicate that the male and female groups differ in OI. However, the various age groups (combination of men and female) indicated almost the same OI as there were no significant results. This particular outcome is in contrast with the findings of both Igbigbi and Ebite [8] and Ezeuko and Om’Iniabohs [7].

Meanwhile, this study suggests a variation between the OI of the same age group when the two gender groups are compared. This is in line with the findings of Igbigbi and Ebite [8] which indicated that within the same age group, female orbital indices were higher than males. This implies that among patients who reported at the CT unit for head CT, the female have higher OI irrespective of their age group.

Finally, ethnic differences with respect to the orbital indices would have been an area of interest to explore; however, as a limitation of the study, this was not included because there was no data on the patients’ ethnic groupings in the storage system of the CT unit.

## Conclusion

The orbital index of Ghanaians as seen in this study is 81.22 ± 4.22. This places the Ghanaians who reported at the CT Unit of KBTH for head CT in the Microseme category of orbit irrespective of their age or gender. This study also showed sexual dimorphism among the study population as the male and female OI were found to be significantly different. In addition, the study for the first time provides baseline information and an anthropomorphic data on the orbit of the Ghanaian population. Thus, the result of this study may be useful in forensic science for skull identification of unknown victims at crime scenes. It may also be useful during planning in surgery. This is very essential for better surgical approach and outcome.

## Abbreviations

CT: Computed tomography
KBTH: Korle-Bu Teaching Hospital
OH: Orbital height
OI: Orbital index
OW: Orbital width
